# Comparing the evidence for botulinum neurotoxin injections in paediatric anterior drooling: a scoping review

**DOI:** 10.1007/s00431-023-05309-1

**Published:** 2023-11-04

**Authors:** Lynn B. Orriëns, Karen van Hulst, Jan J. W. van der Burg, Frank J. A. van den Hoogen, Michèl A. A. P. Willemsen, Corrie E. Erasmus

**Affiliations:** 1 Department of Paediatric Neurology, Division of Paediatrics, Donders Institute for Brain, Cognition and Behaviour, Amalia Children’s Hospital, Radboud University Medical Center, Nijmegen, the Netherlands; 2 Department of Rehabilitation, Donders Institute for Brain, Cognition and Behaviour, Amalia Children’s Hospital, Radboud University Medical Center, Nijmegen, the Netherlands; 3 Department of Paediatric Rehabilitation, Sint Maartenskliniek, Ubbergen, the Netherlands; 4 School of Pedagogical and Educational Science, Radboud University Nijmegen, Nijmegen, the Netherlands; 5 Department of Otorhinolaryngology and Head and Neck Surgery, Radboud University Medical Center, Nijmegen, the Netherlands

**Keywords:** Anterior drooling, Neurodevelopmental disability, Botulinum neurotoxin type-A, Paediatrics, Clinical heterogeneity, Scoping review

## Abstract

Paediatric anterior drooling has a major impact on the daily lives of children and caregivers. Intraglandular botulinum neurotoxin type-A (BoNT-A) injections are considered an effective treatment to diminish drooling. However, there is no international consensus on which major salivary glands should be injected to obtain optimal treatment effect while minimizing the risk of side effects. This scoping review aimed to explore the evidence for submandibular BoNT-A injections and concurrent submandibular and parotid (i.e. four-gland) injections, respectively, and assess whether outcomes could be compared across studies to improve decision making regarding the optimal initial BoNT-A treatment approach for paediatric anterior drooling. PubMed, Embase, and Web of Science were searched to identify relevant studies (until October 1, 2023) on submandibular or four-gland BoNT-A injections for the treatment of anterior drooling in children with neurodevelopmental disabilities. Similarities and differences in treatment, patient, outcome, and follow-up characteristics were assessed. Twenty-eight papers were identified; 7 reporting on submandibular injections and 21 on four-gland injections. No major differences in treatment procedures or timing of follow-up were found. However, patient characteristics were poorly reported, there was great variety in outcome measurement, and the assessment of side effects was not clearly described.

*Conclusion*: This review highlights heterogeneity in outcome measures and patient population descriptors among studies on paediatric BoNT-A injections, limiting the ability to compare treatment effectiveness between submandibular and four-gland injections. These findings emphasize the need for more extensive and uniform reporting of patient characteristics and the implementation of a core outcome measurement set to allow for comparison of results between studies and facilitate the optimization of clinical practice guidelines.

**Table:** 

**What is Known:** *• There is no international consensus on which salivary glands to initially inject with BoNT-A to treat paediatric drooling.*	
**What is New:** *• Concluding on the optimal initial BoNT-A treatment based on literature is currently infeasible. There is considerable heterogeneity in outcome measures used to quantify anterior drooling.and clinical characteristics of children treated with intraglandular BoNT-A are generally insufficiently reported.* *• Consensus-based sets of outcome measures and patient characteristics should be developed and implemented.*

**Supplementary Information:**

The online version contains supplementary material available at 10.1007/s00431-023-05309-1.

## Introduction

First proposed as a paediatric treatment option in 2001 [1], intraglandular botulinum neurotoxin type A (BoNT-A) injections have been widely recognised as an effective intervention for drooling in children with neurodevelopmental disabilities [2]. Drooling may substantially impact daily life for children and caregivers [3, 4], and striving for an optimal treatment approach to diminish drooling is essential. An international consensus statement and care pathway on the treatment of drooling in children have been published in 2010 and 2016, respectively, to enhance patient care [2, 5]. However, no recommendations were offered regarding which salivary glands should initially be injected with BoNT-A. Consequently, clinicians caring for these patients currently lack guidance in making this decision.

Saliva is mainly produced by three major bilateral salivary glands: the parotid, submandibular and sublingual glands. In rest, the submandibular salivary glands are responsible for the majority (approximately 70%) of saliva production [6]. Consequently, it has been hypothesised that BoNT-A injections into the submandibular glands would sufficiently reduce salivary flow to diminish drooling, whilst preserving the secretion of saliva from the parotid glands during mastication [1, 6–10]. Especially in children with chewing difficulties (e.g. inefficient chewing or a munching chewing pattern), parotid saliva is considered essential to sufficiently moisten solid foods.

Internationally, concurrent BoNT-A injections into the submandibular and parotid glands (i.e. four-gland injections) are commonly used as well. Several authors hypothesise that diminishment of both stimulated saliva production and resting saliva production would be essential to successfully reduce drooling [11–14]. In our saliva control clinic, a stepped care approach has been implemented; submandibular injections are administered initially, whereas four-gland injections are generally reserved for children with a clinically insufficient response to submandibular injections. This raises the question whether our initial treatment approach is too cautious (i.e. resulting in undertreatment) or whether using four-gland injections in all children leads to overtreatment, potentially increasing the risk of xerostomia and problems processing solid foods.

The existing literature on this topic could be used to address this question, by comparing the outcomes of studies on submandibular injections to those of studies on four-gland injections in a systematic review and meta-analysis. However, this would only be feasible if sufficient literature is available and in the absence of significant differences across these studies in characteristics that may influence reported effectiveness or negative effects of the treatment. These differences could comprise variation in treatment procedures, patient characteristics, outcome measures, or timing of follow-up (i.e. clinical heterogeneity) [15].

Despite the availability of the aforementioned guidelines, which primarily aimed to optimise patient care, there may be substantial international disparities in how research on the effectiveness of BoNT-A for paediatric drooling is conducted and reported, which patients are included, and how they are treated. Therefore, we aimed to scope the literature, exploring available evidence on either submandibular or four-gland BoNT-A injections and assessing similarities and differences in the aforementioned characteristics across studies. We strived to ascertain whether results could be compared effectively, ultimately aiding decision-making regarding the initial choice of salivary glands to be injected.

## Methods

A scoping review was performed to map the available evidence on both treatment approaches and evaluate the comparability of treatment, patient, outcome, and follow-up characteristics. Rather than summarize a relatively narrow range of quality-assessed studies, scoping reviews are designed to describe research findings and the range of research in more detail, and identify gaps in the evidence that should be addressed in future studies [16, 17].

For the methodology of this scoping review, we adhered to the commonly-used framework suggested by Arksey and O’Malley [17] and updated by Levac et al. [18]. The review was written in accordance with the Preferred Reporting Items for Systematic Reviews and Meta-Analyses Extension for Scoping Reviews [19]. The five key methodological components of this review (i.e. the research question, search strategy, study selection process, eligibility criteria, and data charting process) were outlined in an a priori review protocol, which was not made publicly available.

### Research question

We aimed to assess whether existing literature may be used to conclude on the optimal initial approach for BoNT-A injections. Therefore, the review was guided by two questions:

1. What is the extent and nature of published scientific literature on the effectiveness of either submandibular or four-gland BoNT-A injections in children with drooling secondary to neurodevelopmental disabilities?
2. To what extent are studies on each treatment approach comparable with regard to treatment procedures, patient characteristics, outcome measures, and timing of follow-up?

### Data sources and search strategy

We systematically searched 3 electronic databases (PubMed, Embase, and Web of Science). The following elements were used to build a comprehensive search strategy: children AND neurodevelopmental disabilities AND botulinum toxin type-A injections AND drooling, and their synonyms. No limits were set for publication date. Search strategies can be found in Supplemental Table 1. A final search in all three databases was conducted on October 1, 2023. Search results were collected and deduplicated in Endnote [20].

### Study selection

Eligibility criteria are detailed in Table 1. The reference lists of relevant excluded (systematic) reviews and meta-analyses were manually screened to assess the presence of uncaptured studies.

**Table 1:** Eligibility criteria

**Inclusion criteria**	**Exclusion criteria**
Studies with empirical data	Non-empirical studies (e.g. clinical summaries, opinion pieces, grey literature)
Published in English, German, or Dutch	Meta-analyses or systematic reviews
Includes children and/or adolescents (0–21 years) diagnosed with neurodevelopmental disability and anterior drooling	Only one patient evaluated
Intervention consisting of bilateral BoNT-A injections in the submandibular salivary glands, or concurrent injections in the submandibular and parotid salivary glands to treat anterior drooling	No outcomes with regard to effectiveness reported
	Both treatment approaches assessed, and patient characteristics and/or results not reported separately
	Selective patient population (i.e. inclusion dependent on previous or subsequent treatment)
	Substantial overlap in patient cohorts (i.e. studies published by the same research team, with overlap in inclusion period)

The initial screening of article titles and abstracts and subsequent full-text review of all remaining studies were performed by the first and second author, independently. Discrepancies between both reviewers were resolved by discussion and consensus. No methodological quality assessment was performed [17].

### Data charting

A data extraction form, collectively developed by the research team, was used to chart data from the included studies. Extracted data comprised:

- Study characteristics (i.e. study design, sample size, year(s) of inclusion)
- Treatment procedures (i.e. BoNT-A formulation and dose, use of ultrasound guidance and anaesthesia)
- Patient characteristics (i.e. demographic and clinical characteristics)
- Type (i.e. objective/subjective) and construct (e.g. drooling severity, impact of drooling, quality of life) of outcome measures used to quantify treatment effect
- Timing of follow-up assessments after BoNT-A treatment
- Assessment of negative effects (i.e. side effects and adverse effects)

Data were charted by the first author and summarized in a descriptive table, stratified for treatment approach. The corresponding author of one included study [21] was contacted to verify data on negative effects.

### Research team

The research team for this scoping review was composed of a speech-language therapist/clinical epidemiologist (KH), two paediatric neurologists (CE and MW), a biomedical scientist (LO), a healthcare psychologist (JB), and an otorhinolaryngologist/head and neck surgeon (FH). The majority of the authors are part of the multidisciplinary saliva control team at the Radboud University Medical Centre (Radboudumc) Amalia Children’s Hospital in Nijmegen, the Netherlands.

## Results

### Search and selection of relevant studies

**Fig. 1:**
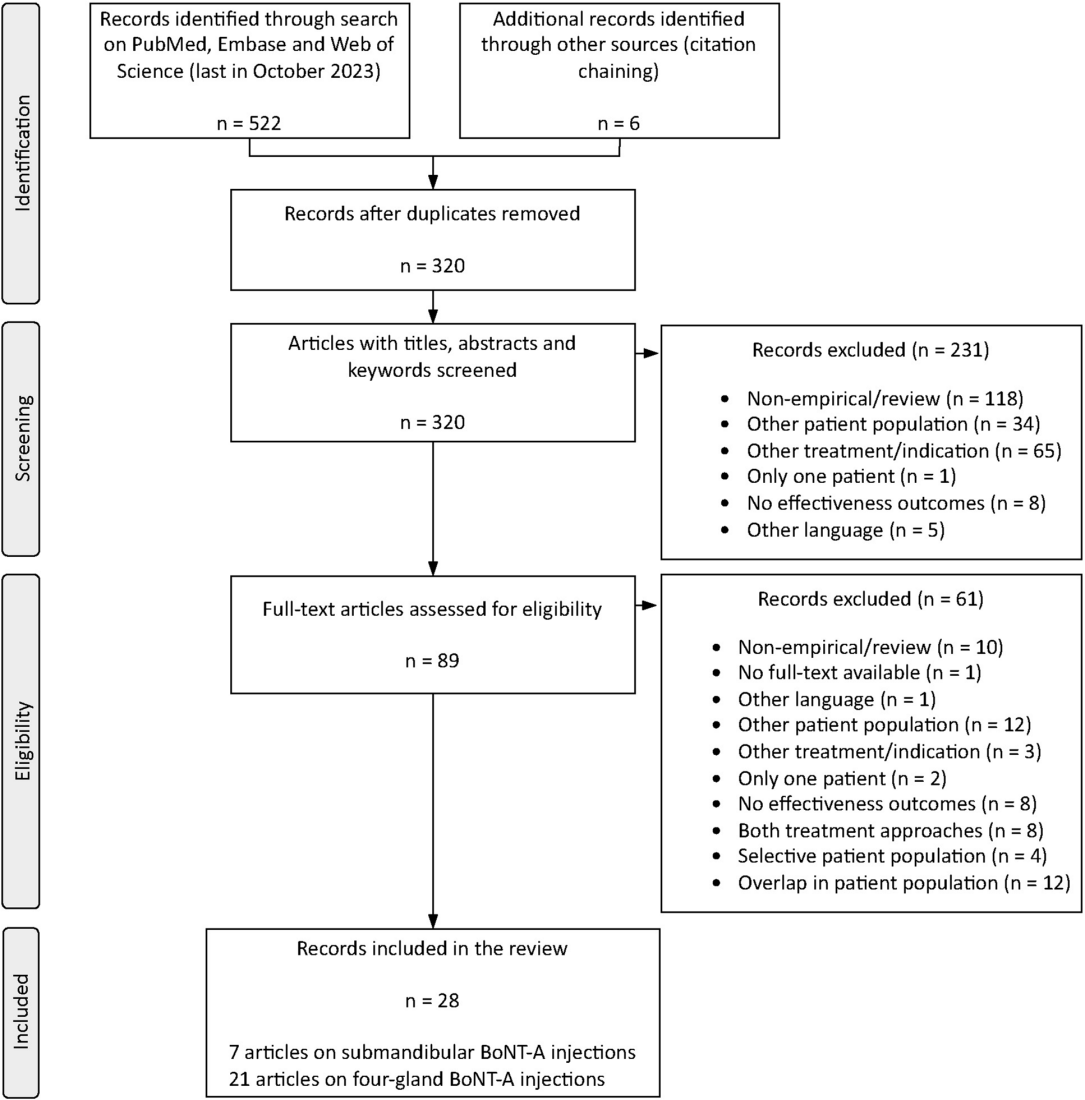
Flowchart representing the process of study selection and inclusion

The flow of articles from identification through inclusion is presented in Fig. 1. A final search in October 2023, after deduplication, yielded 320 potentially relevant articles. Finally, 28 articles reporting on 27 unique studies were included in the review. Five of these studies included a small proportion of patients aged ≥ 21 years [9, 22–25]. The decision was made to nevertheless include them, considering that the vast majority of the study population fell within the correct age range.

The results discussed in the following paragraphs are further elucidated in Supplemental Table 2.

### Characteristics of literature on effectiveness of BoNT-A injections

Table 2 summarizes the characteristics of the 27 studies included in this review, stratified by treatment approach. Less studies on submandibular BoNT-A injections (*n* = 6) were included than on four-gland injections (*n* = 21). The majority of studies were prospective, though studies on submandibular injections were more often retrospective in nature than studies on four-gland injections (50% vs 14%, respectively).

**Table 2:** Characteristics of included studies, stratified by treatment approach

	Submandibular injections, *n* (%)		Four-gland injections, *n* (%)	
Number of included studies	6 (100)		21 (100)	
Study methodology				
Retrospective	3 (50)		3 (14)	
Prospective	3 (50)		18 (86)	
Years of publication, *range*	2011–2020		2006–2023	
Treatment characteristics				
BoNT-A formulation				
Botox^®^	6 (100)		14 (67)	
Dysport^®^	0 (0)		1 (5)	
Xeomin^®^	0 (0)		2 (10)	
Not reported	0 (0)		4 (19)	
Dose within range	6 (100)		20 (95)	
Ultrasound guidance	5 (83)		18 (86)	
Anaesthesia (i.e. sedation or general anaesthesia)	5 (83)		15 (71)	
Outcome measures				
Type				
Objective only	0 (0)		0 (0)	
Subjective only	3 (50)		15 (71)	
Both objective and subjective	3 (50)		6 (29)	
Construct				
Drooling frequency/severity only	3 (50)		11 (52)	
Impact/QoL only	0 (0)		0 (0)	
Both frequency/severity and impact/QoL	3 (50)		10 (48)	
Reporting of patient characteristics^a^				
Age	6 (100)		20 (95)	
Diagnosis	6 (100)		20 (95)	
Subtype^b^ in case of CP diagnosis	2 (23)		4 (19)	
Epilepsy	2 (33)		9 (43)	
GORD	2 (33)		2 (10)	
Cognitive abilities	2 (33)		4 (19)	
Posture/head control	0 (0)		2 (10)	
Ambulation level	4 (67)		10 (48)	
Feeding abilities	2 (33)		7 (33)	
Speech abilities	1 (17)		4 (19)	
Baseline drooling severity	5 (83)		17 (81)	

*BoNT-A* botulinum neurotoxin type-A, *CP* cerebral palsy, *GORD* gastro-oesophageal reflux disease, *QoL* quality of life

^a^Frequencies and percentages represent the number of studies that reported on each specific patient characteristic

^b^Spastic, dyskinetic, ataxic, or mixed

The median sample size of studies concerning submandibular injections (*n* = 23, IQR: 50) was similar to that of studies on four-gland injections (*n* = 20, IQR: 15). In total, the included studies reflected a population of 250 patients and 700 patients for submandibular and four-gland injections, respectively.

### Comparability of studies on each treatment approach

#### Treatment procedures

All reviewed studies on submandibular BoNT-A injections used Botox^®^. The administered dose ranged between 15 and 25 U per gland, which was within the recommended range (i.e. 10–50 U per gland) [5]. Three studies used a bodyweight-dependent dose [26–28].

Although Botox^®^ was used most commonly among studies on four-gland injections as well (*n* = 14), two studies used Xeomin^®^ [24, 29], and one study used Dysport^®^ [30]. In twelve studies, bodyweight- or age-dependent doses were used. Administered doses were within the recommended range (i.e. 10–50 U per gland for Botox^®^, 20–75 U in total for Xeomin^®^, and 15–75 U per gland for Dysport^®^) [5, 31], except for one study where a total dose of 150 U of Xeomin^®^ was used [24].

Most included studies, across both treatment approaches, used ultrasound guidance to ensure accurate delivery of the injected BoNT-A into the salivary glands. Sedation or general anaesthesia was used in all but one (83%) of the studies on submandibular injections, whereas only fifteen studies (71%) concerning four-gland injections administered anaesthesia in a majority of the patients.

#### Patient characteristics

On average, included children were most often diagnosed with cerebral palsy (mean percentage of 56% in studies on submandibular injections, 69% in studies on four-gland injections). One study on submandibular injections and four studies on four-gland injections included some patients with mild drooling [25, 26, 32–34]. Otherwise, baseline drooling was classified as (moderate to) severe according to the Drooling Severity and Frequency Scale (DSFS) or Teacher Drool Scale (TDS).

Only half of the studies reported ambulation level. Within these studies, the proportion of non-ambulant children and children with gross motor function classification system (GMFCS) levels IV to V (i.e. children with the most severe degree of motor impairment) differed extensively. Other distinctive patient characteristics, including cerebral palsy subtype, comorbidities (i.e. epilepsy and gastro-oesophageal reflux disease [GORD]), cognitive abilities, posture or head control, feeding ability, and speech ability were poorly reported.

#### Outcome measures and treatment response

In addition to the frequency and/or severity of drooling, which was a construct evaluated in all included studies, the impact of drooling on daily life or quality of life were assessed in 13 studies (48%). A combination of objective and subjective outcome measures was used in half of the studies on submandibular injections, whereas the majority of studies on four-gland injections (70%) reported only subjective outcome measures.

Closer examination revealed 26 distinct outcome measures applied across the included studies, of which 12 were existing metrics and 14 were self-constructed instruments. Most frequently used measures were the Drooling Severity and Frequency Scale (DSFS, *n* = 14) [35], drooling quotient (DQ, *n* = 6) [36, 37], Visual Analogue Scale (VAS) for drooling severity and/or frequency (*n* = 6) [38], and salivary flow rate (*n* = 5). Less than half of the included studies applied at least one of the same outcome measures.

The proportion of children who responded to treatment (i.e. response rate) was evaluated in 67% and 76% of studies on submandibular and four-gland injections, respectively, whereas the remaining studies only described mean or median baseline and post-injection scores. The criteria used to define treatment response differed greatly across studies (Supplemental Table 3). All studies on submandibular injections that reported response rates used (a variety of) cut-off limits to define a meaningful or clinically significant change compared to baseline, whereas this was the case for merely 44% of studies on four-gland injections.

#### Timing of follow-up

Although the timing and frequency of follow-up varied substantially across studies, most studies (83% and 81% of studies on submandibular injections and four-gland injections, respectively) reported the results from at least one follow-up measurement between 4 and 8 weeks post-procedure. This timing is considered adequate for the assessment of treatment effect when taking response latency [39–41], peak improvement time [1, 34, 38, 42], and duration of effect into account [43]. Long-term effects (≥ 20 weeks post-injection) were evaluated in 50% of the studies on submandibular injections and 19% of the studies on four-gland injections.

#### Assessment of negative effects

To conclude on the optimal initial treatment approach, the occurrence of negative effects (i.e. side effects and adverse effects) and the extent to which these are assessed should be taken into account. Among the reviewed studies, the reported side effect and adverse effect rate ranged from 5 to 15% (median: 11%) for studies on submandibular injections and 0 to 78% (median: 9%) for studies on four-gland injections. Characteristics of all reported negative effects are detailed in Supplemental Table 2. Nevertheless, it was generally not clear when (i.e. systematically or on indication) and how (i.e. using a structured symptom list or an open-ended question) negative effects were assessed.

## Discussion

This scoping review highlights that existing literature does not provide clear guidance on the optimal number of salivary glands to target in a first BoNT-A treatment session for drooling in children with neurodevelopmental disabilities. The lack of standardization in patient population descriptions, outcome measures, and criteria for treatment response among the included studies makes it impossible to draw accurate comparisons between their results.

The current review demonstrates that there is substantial evidence available for both submandibular BoNT-A injections and four-gland BoNT-A injections, albeit with a greater number of studies covering the latter treatment approach. The sample sizes in the included studies varied, but the median number of children included was comparable for both treatment approaches. Furthermore, almost all included studies adhered to the recommended dosing of BoNT-A and a majority employed ultrasound guidance to ensure accurate administration of the injections. These findings suggest a global consensus in treatment procedures and conformity to established guidelines.

Nevertheless, our findings highlight significant diversity in outcome measures used to quantify the effectiveness of BoNT-A injections, with a total of 26 different scales and questionnaires being applied. Moreover, treatment response rate (i.e. the proportion of children who demonstrated an adequate response to treatment, as a binary outcome variable) was not consistently reported and the definition of treatment response varied considerably. It is important to note that a minor reduction in drooling severity may not translate to meaningful differences in the child’s and their family’s lives [3, 44], which will also affect treatment satisfaction. Hence, cut-off limits to indicate satisfactory response should be carefully chosen.

The optimal method to quantify drooling has been a topic of debate for many years [45]. A range of (semi-)objective and subjective (i.e. caregiver-reported) outcome measures are available to assess the severity and frequency of drooling and, consequently, treatment effectiveness [46]. Additionally, it is important for clinicians to consider the impact of drooling on the child’s life and that of their caregivers. While most studies in this scoping review recognized the significance of incorporating caregiver-reported outcome measures, only half of them assessed the impact of drooling or the child’s quality of life and few studies included this construct in their definition of treatment response.

To enable a comparison of outcomes across available literature in the future, harmonisation of outcome measurement is the first hurdle to take. This could be addressed through the development and implementation of a consensus-based, standardized set of outcome measures to be measured and reported in future studies on anterior drooling in children, known as a core outcome measurement set (COMS) [47]. Ensuring uniformity in measurement, a COMS enables direct comparison of results across studies, increasing the feasibility of conducting systematic reviews and meta-analsyes [48, 49]. Additionally, by involving a wide range of stakeholders (e.g. patients, caregivers, and health care professionals) during its development, the reporting of clinically relevant outcomes is stimulated [50].

Based on the various outcome measures used in the studies included in this scoping review and supported by our clinical experience, we would consider it important to include both objective and caregiver-reported measurements of the severity and frequency of drooling in this set [37]. Additionally, it is imperative to assess how drooling affects the daily lives of both children and caregivers. Nonetheless, it is important to carefully consider the feasibility of reporting different outcome measures across various study designs. To capture the child’s perspective, the use of existing outcome measures for drooling severity, frequency, and its impact may be feasible in children who are able to self-report, potentially requiring minor adjustments in phrasing and the inclusion of age-appropriate response options [51]. Ideally, children should also be involved during the COMS development process as stakeholders [52]. In cases where children are unable to self-report as a result of their cognitive and/or communicative abilities or age, caregivers will remain vital advocates. It is important to explicitly address how drooling affects the child’s life, encompassing not only practical and physical consequences but also exploring the social and emotional implications, even when children may not be aware of their drooling. The implementation of child-centred outcome measures, aimed at capturing the impact of drooling on each individual child and family, would eventually be a valuable asset [53]. Updating or expanding of a COMS over time is stimulated to keep the set up-to-date with advances in outcome measurement [54].

Nevertheless, even if the same set of outcome measures would have been used in the included studies, patient characteristics were not reported extensively enough to ensure the comparability of study populations. As suggested previously, the development of a consensus-based standardised set of patient characteristics that should consistently be reported (i.e. a core descriptor set) should be integrated in the process of developing a COMS [55]. We would recommend that characteristics that may influence the effectiveness of treatment or susceptibility to side effects should at least be documented, including the child’s age, diagnosis and co-morbidities (e.g. epilepsy and GORD) [33, 56–61], cognitive abilities [62–64], posture or head control [5, 28, 65–67], ambulation level [35, 68], oral motor control [65, 69, 70], eating abilities [71], and baseline drooling severity. Standardised reporting of these characteristics will enable an assessment of clinical heterogeneity and will allow systematic reviewers to adjust for these differences (e.g. through pre-specified subgroup analyses) when comparing outcomes [72]. A balance should be sought between the number of characteristics included in the set to allow for a robust synthesis of studies and potential challenges involved in data collection [55].

Finally, a (multicentre) comparative effectiveness study, applying these consensus-based sets of outcome measures and patient characteristics, could help to further explore the relative effectiveness of submandibular versus four-gland BoNT-A injections. Besides being able to assess whether one of both approaches is more effective overall, relative to the risk of negative effects, a comparative study could focus on inter-individual differences in treatment response. This could guide personalized treatment decisions and eventually improve the cost-effectiveness of BoNT-A treatment [73, 74].

### Strengths and limitations

To our knowledge, this was the first review comparing evidence for submandibular and four-gland BoNT-A injections. Robust search terms were used, which ensured a comprehensive and systematic search of the literature, and eligibility assessment was conducted by two authors. However, this review is not without limitations. First, some relevant studies might have been missed by excluding grey literature [75] and languages other than English, German or Dutch, as well as by limiting our search to three biomedical literature databases. Second, no critical appraisal was applied to the included studies. Nevertheless, we aimed to map all available evidence on BoNT-A injections for paediatric drooling and assess its comparability between two treatment approaches, to which an assessment of the strength of the evidence was not expected to contribute. Moreover, a methodological quality assessment is generally not part of the scoping review approach [17, 18]. Third, we focused on the comparison between submandibular and four-gland injections, whereas other combinations of glands may be targeted as well (e.g. parotid gland injections [76–79], one submandibular and one parotid gland injected [80]).

## Conclusions

This scoping review concludes that it is infeasible to determine the optimal initial approach for BoNT-A injections in the treatment of paediatric drooling based on existing literature. The study identified a significant lack of consistency in outcome measures, descriptors of patient characteristics, and criteria used to define treatment response among published studies on submandibular and four-gland BoNT-A injections. Recommendations to optimise research practices and eventually patient care are summarised in Table 3. It is imperative that a consensus-based, uniform set of core outcome measures and patient descriptors be implemented internationally. This will allow for more reliable comparisons of studies on treatment for paediatric drooling in the future and contribute to more effective decision-making in clinical practice.

**Table 3:** Recommendations for future research on interventions for paediatric anterior drooling

Comprehensive data collection and reporting	
To assess and report	Patient characteristics that may influence the effectiveness of treatment or susceptibility to side effects, providing a comprehensive overview of the patient population.
	The impact of (anterior) drooling on (quality of) life, both from the child- and caregiver perspective, in addition to its severity and frequency.
	The proportion of children that responded adequately to treatment (i.e. treatment response rate) to reflect its effectiveness at patient level.
	Side effects and their timing relative to the intervention in a standardised manner

Development of core measurement and descriptor sets	
To develop	A core outcome measurement set (COMS), preferably including both objective and caregiver-reported outcome measures on drooling severity and frequency, as well as an assessment of the impact of drooling on daily life.
	A core descriptor set (CDS), reflecting a set of patient characteristics that provide an adequate overview of the patient population and can be accounted for when establishing treatment effectiveness and/or side effects.
To establish	Consensus on a cut-off limit for ‘treatment response’ based on the outcome measures included in the COMS.

Comparative effectiveness study	
To conduct	A comparative effectiveness study on submandibular and four-gland BoNT-A injections, which can be used to identify inter-individual differences in treatment response in addition to providing insight into the overall comparison of both treatment approaches.

### Supplementary Information

Below is the link to the electronic supplementary material.

Supplementary file1 (DOCX 82 KB)
