# Supplementary material for: Comparing the evidence for botulinum neurotoxin injections in paediatric anterior drooling: a scoping review
Source: Eur J Pediatr. 2023 Nov 4;183(1):83–93. doi: 10.1007/s00431-023-05309-1 (PMC10858158; doi:10.1007/s00431-023-05309-1)
Supplement: Supplementary file 1 — Supplementary file1 (DOCX 82 KB) [file 431_2023_5309_MOESM1_ESM.docx]

**Table S1**. Search strategies to identify studies in which the effectiveness of the use of BoNT-A injections to treat drooling in children with neurodevelopmental disorders is evaluated.

| **PubMed** |
| --- |
| (((((((((((child*[Title/Abstract]) OR infant*[Title/Abstract]) OR pediatric[Title/Abstract]) OR Child[MeSH Terms]) OR Child, Preschool[MeSH Terms]) OR Infant[MeSH Terms]) OR Adolescent[MeSH Terms]) OR Pediatrics[MeSH Terms])) AND  (((((((((neurodevelopmental dis*[Title/Abstract]) OR neurological dis*[Title/Abstract]) OR developmental dis*[Title/Abstract]) OR intellectual dis*[Title/Abstract]) OR mental dis*[Title/Abstract]) OR cerebral palsy[Title/Abstract]) OR Neurodevelopmental disorders[MeSH Terms]) OR Developmental disabilities[MeSH Terms]) OR Cerebral Palsy[MeSH Terms])) AND  ((((((((botulinum toxin injection*[Title/Abstract]) OR botox injection*[Title/Abstract]) OR botulinum toxin[Title/Abstract]) OR botulinum neurotoxin[Title/Abstract]) OR onabotulinumtoxinA[Title/Abstract]) OR incobotulinumtoxinA[Title/Abstract]) OR abobotulinumtoxinA[Title/Abstract]) OR Botulinum Toxins, Type A[MeSH Terms])) AND  (((((((drool*[Title/Abstract]) OR hypersalivation[Title/Abstract]) OR dribbling[Title/Abstract]) OR sialorrhea[Title/Abstract]) OR saliva*[Title/Abstract]) OR saliva control[Title/Abstract]) OR Sialorrhea[MeSH Terms]) |
| **Embase** |
| (child*.ti,ab,kf. OR infant*.ti,ab,kf. OR paediatric.ti,ab,kf. OR pediatric.ti,ab,kf. OR child/ OR preschool child/ OR school child/ OR infant/ OR toddler/ OR adolescent/ OR pediatrics/) AND  (neurodevelopmental dis*.ti,ab,kf. OR developmental dis*.ti,ab,kf. OR intellectual dis*.ti,ab,kf. OR mental dis*.ti,ab,kf. OR neurological dis*.ti,ab,kf. OR cerebral palsy.ti,ab,kf. OR mental disease/ OR developmental disorder/ OR cerebral palsy/) AND  (botulinum toxin injection*.ti,ab,kf. OR botox injection*.ti,ab,kf. OR botulinum toxin.ti,ab,kf. OR botulinum neurotoxin.ti,ab,kf. OR onabotulinumtoxinA.ti,ab,kf. OR incobotulinumtoxinA.ti,ab,kf. OR abobotulinumtoxinA.ti,ab,kf. OR botulinum toxin A/) AND  (hypersalivation.ti,ab,kf. OR dribbling.ti,ab,kf. OR sialorrhea.ti,ab,kf. OR drool*.ti,ab,kf. OR saliva*.ti,ab,kf. OR saliva control.ti,ab,kf. OR hypersalivation/) |
| **Web of Science** |
| (child* OR infant* OR paediatric OR pediatric) AND  (neurodevelopmental dis* OR neurological dis* OR developmental dis* OR intellectual dis* OR mental dis* OR cerebral palsy) AND  (botulinum toxin injection* OR botox injection* OR botulinum toxin OR botulinum neurotoxin OR onabotulinumtoxinA OR incobotulinumtoxinA OR abobotulinumtoxinA) AND  (drool* OR hypersalivation OR dribbling OR sialorrhea OR saliva* OR saliva control) |

**Table S2.** Summary of included studies

| **First author, location, and year published** | **Study design and year(s) of inclusion** | **Sample size and characteristics of study population** | **Treatment procedures (ultrasound guidance, anaesthesia, BoNT-A formulation and dose)** | **Timing of follow-up (reported)** | **Outcome measures** | **Reported patient character-istics** | **Adverse effect rate and specification** |
| --- | --- | --- | --- | --- | --- | --- | --- |
| **Submandibular gland injections** | | | | | | | |
| Bekkers^1^  (Nijmegen, the Netherlands), 2019 | RCT‡ (BoNT-A vs. submandibular duct ligation)  2012-2017 | 26 children; 15 boys, 11 girls  Aged 8-22 years (mean age 11y 2m; SD 2y 6m)  Primary diagnosis: spastic CP (n=10), dyskinetic CP (n=1), spastic/ dyskinetic CP (n=5), CP not further specified (n=1), other non-progressive neurodevelopmental disability (n=9)  All children had severe drooling (DF and DS score ≥3 and ≥2, respectively) at baseline | UG,  general anaesthesia  Botox®  25 U/gland | Baseline,  8 weeks,  32 weeks | Objective (DQ*)  Subjective (VAS*) | Age  Diagnosis  + CP subtype  Epilepsy  GORD  Feeding  Cognition  Mobility  Baseline drooling | 4 (15%)  Dysphagia (n=2), diminished feeding due to nausea (n=1), pneumonia (n = 1) |
| Bekkers^2^  (Nijmegen, the Netherlands), 2021 |  |  |  |  | Questionnaire on impact* |  |  |
| Ciftci^3^  (Ankara, Turkey), 2013 | Retrospective cohort study  2006-2011 | 20 children; 15 boys, 5 girls  Aged 3-16 years (mean age 9.1 years)  Primary diagnosis: CP (n=8), motor and mental retardation (n=4), hypoxic ischemic encephalopathy (n=2), other underlying disease (n=6)  All children had severe drooling (TDS score ≥3) at baseline | UG, intravenous sedation  Botox®  15U/gland for <15 kg, 20 U/gland for 15-25 kg, 25 U/gland for >25 kg – mean dose 20U/gland | Baseline,  4 weeks,  12 weeks | Subjective (TDS*) | Age  Diagnosis  Baseline drooling | 1 (5%)  Intermittent problems with swallowing due to viscous saliva (n=1) |
| Gok^4^  (East Sussex, UK), 2013 | Prospective cohort study  2008-2011 | 9 children; 6 boys, 3 girls  Aged 3-25 years (mean age 12 years)  Primary diagnosis: CP (n=1), severe learning disability (n=8)  All children had severe drooling (DS and DF score ≥4 and 4, respectively) at baseline | UG,  general anaesthesia  Botox®  20-25U/gland | Baseline,  6 weeks | Subjective (DSFS*) | Age  Diagnosis  Baseline drooling | 1 (11%)  Difficulty keeping prosthetic globe in place (n=1) |
| Hay^5^ (South Africa), 2011 | Retrospective cohort study  Not reported | 9 children; 6 boys, 3 girls  Aged 5-17 years (mean age 9y 4m)  Primary diagnosis: spastic CP (n=2), dyskinetic CP (n=3), ataxic CP (n=2), operculum syndrome (n=2)  7 children had severe drooling (DS score ≥4), 2 children had mild drooling (DS score 2) at baseline | Manual manipulation, anaesthesia not reported  Botox®  15-25U/gland dependent on body weight | Baseline,  8 weeks, 24-26 weeks | Objective (drooling severity based on photographs in several situations)  Subjective (DSFS*)  Questionnaire on impact | Age  Diagnosis  + CP subtype  GORD  Speech  Mobility  Baseline drooling | Not reported |
| Mahadevan^6^ (New Zealand), 2016 | Retrospective chart review  2008-2013 | 26 children† (48 procedures); 14 boys, 12 girls  Aged 7 months-18 years (mean age 9.5 years) at time of injection  Primary diagnosis: CP (n=17), other neurodevelopmental disability (n=9) | UG,  general anaesthesia  Botox®  15-25U/gland dependent on body weight | Baseline,  3 months | Subjective (DSFS*) | Age  Diagnosis  Mobility | Not reported |
| van Hulst^7^ (Nijmegen, the Netherlands), 2020 | Prospective, observational study  2000-2012 | 160 children; 92 boys, 68 girls  Aged 3-17 years (mean age 9y 1m; SD 3y 6m)  Primary diagnosis: CP (n=123), other non-progressive neurodevelopmental disability (n=37)  All patients had severe drooling (TDS score ≥3) at baseline | UG,  general anaesthesia  Botox®  25U/gland | Baseline,  8 weeks,  32 weeks | Objective (DQ5*)  Subjective (VAS*)  Questionnaire on impact* | Age  Diagnosis  Epilepsy  Feeding  Cognition  Mobility  Baseline drooling | Not reported |
| **Four-gland injections** | | | | | | | |
| Alvarenga^8^ (Portugal), 2017 | Prospective cohort study  2012-2015 | 17 children† (25 procedures); 11 boys, 6 girls  Aged 4-19 years (mean age 12.1 years; SD 5.1 years)  Primary diagnosis: hypoxic-ischemic syndromes (n=12), rare syndromes (n=5)  All children had severe drooling (DS and DF score ≥4 and ≥3, respectively) at baseline | UG,  general anaesthesia  Botox®  30U/parotid gland, 20U/submandibular gland | Baseline,  1 month,  3 months,  6 months | Subjective (DSFS*) | Age  Diagnosis  Baseline drooling | 1 (4% of all procedures)  Mild transient dysphagia (n=1) |
| Banerjee^9^ (Sydney, Australia), 2006 | Descriptive open-label prospective study  Not reported | 20 children; 10 boys, 10 girls  Aged 6y 1m-16y 7m (mean age 10y 1 m; SD 3y 8m)  Primary diagnosis: spastic CP (n=17), dystonic CP (n=1), mixed CP (n=2)  All children had severe drooling (DSFS score ≥6) at baseline | UG,  topical anaesthetic and sedation  Botox®  2U/kg divided over the four glands | Baseline,  4 weeks,  12 weeks | Objective (saliva weight*, DQ*)  Subjective (DSFS*, no. bibs/day*)  QoL questionnaire | Age  Diagnosis  + CP subtype  Mobility  Baseline drooling | 2 (10%)  Transiently dry lips (n=1), dry saliva ball attached to palate (n=1) |
| Bernardo^10^ (Italy), 2019 | Prospective cohort study  2017-2018 | 5 children; 0 boys, 5 girls  Aged 13-23 years (mean age 18.4 years; median age 19 years)  Primary diagnosis: Rett syndrome (n=5)  Median DSFS score 4 (IQR 1) at baseline | UG, anaesthesia not reported  Xeomin®  150U divided over the four glands | Baseline,  4 weeks,  12 weeks | Subjective (DSFS*) | Age  Diagnosis  Epilepsy  GORD  Feeding  Speech  Posture Mobility  Baseline drooling | 1 (20%)  Worsened swallowing difficulties 2 weeks after injection, recovering 4 weeks later, not resulting in major complications (n=1) |
| Berweck^11^ (Georgia, Hungary, Poland, Russia, Serbia, Ukraine), 2021 | Multicentre RCT‡ (BoNT-A vs. placebo)  2015-2019 | 183 children across two age-based cohorts (2-5 year-olds and 6-17 year-olds); 115 boys, 68 girls  Aged 2-17 years (mean age 3.9 years; SD 0.9 years in the 2-5 cohort) (mean age 10.4 years; SD 3.2 years in the 6-17 cohort) (overall mean age 9.2 years)  Primary diagnosis: CP (n=122), traumatic brain injury (n=9), other neurologic condition (n=52)  All children had severe drooling (mTDS score ≥6) at baseline | UG,  analgesia and sedation in ±90%  Xeomin®  20-75U divided over the four glands | Baseline,  4 weeks,  8 weeks,  12 weeks | Objective (salivary flow rate*, DQ*)  Subjective (GICS*, mTDS*)  Only averaged DQ and mTDS scores reported | Age  Diagnosis  Cognition  Mobility  Baseline drooling | 32 (18%)  Mild, transient dysphagia (n=1), nasopharyngitis (n=3), administration site conditions (n=1), other adverse effects not specified |

| Ghazavi^12^ (Isfahan, Iran), 2023 | Prospective cohort study | 12 children; 4 boys, 8 girls  Aged 1-16 years (mean age 6.87 years; SD 4.12 years)  Primary diagnosis: CP (n=12)  DSFS score of ≥5 at baseline | UG, general anaesthesia  Botox®  0.5 U/kg/gland | Baseline,  2 weeks,  4 weeks,  8 weeks,  16 weeks, 24 weeks | Subjective (DSFS*, volume score) | Age  Diagnosis  Baseline drooling | 3 (25%)  Dysphagia (n=2), bleeding from injection site (n=1) |
| --- | --- | --- | --- | --- | --- | --- | --- |
| Gubbay^13^ (Perth, Australia), 2019 | Prospective observational case series  2010-2014 | 15 children† (71 procedures); 8 boys, 7 girls  Aged 3-14 years (mean age 9.9 years; SD 3.4 years) at baseline  Primary diagnosis: CP (n=11), Angelman syndrome (n=1), ATRX syndrome (n=1), 1q44 microdeletion (n=1), undiagnosed developmental impairment (n=1)  12 patients had severe drooling (DS and DF score ≥4 and ≥3, respectively) at baseline, 3 patients had moderate drooling (DS score 3) | UG,  topical anaesthetic and sedation or general anaesthesia  BoNT-A formulation not reported  1U/kg/gland | Baseline,  1-3 months after final injection | Subjective (DSFS*)  Impact on daily life | Age  Diagnosis  Feeding  Mobility  Baseline drooling (DSFS) | 1 (1% of all procedures)  Transient unilateral facial weakness, resolving spontaneously within 2 days (n=1) |
| Lungren^14^ (Ohio, USA), 2016 | Retrospective chart review  2004-2014 | 111 children† (144 procedures); 63 boys, 48 girls  Aged 4 months-34 years (mean age 7 years)  Primary diagnosis: CP (n=32), encephalopathy (n=6), anoxic brain injury (n=4), chromosomal anomaly (n=5), other diagnosis (n=64) | UG,  general anaesthesia  BoNT-A formulation not reported  15U/gland for <15 kg, 20U/gland for 15-25 kg, 25U/gland for >25 kg | 2 days to 9 years, no details on timing | Subjective (composite outcome consisting of drooling severity, QoL and improvement relative to expectations, combined with duration of effect) | Age  Diagnosis | 3 (2% of all procedures)  Cellulitis (n=1), temporary unilateral minor muscle weakness manifesting as mastication difficulty, resolving within 4 weeks (n=2) |
| Meece^15^ (Delaware, USA), 2010 | Retrospective cohort study  2005-2007 | 6 children† (14 procedures)  Aged 2-17 years  Primary diagnosis: predominantly CP | UG,  general anaesthesia  BoNT-A formulation not reported  30U/parotid gland, 20U/submandibular gland | Not specified (at least one year after injection) | Subjective ( questionnaire with 25 items on drool reduction, need for suctioning, bib changes, respiratory distress, QoL, complications) | Age range  Predominant diagnosis | 1 (7% of all procedures)  Very small amount of transient swelling around the injection site (n=1) |
| Montgomery^16^ (Glasgow, UK), 2014 | Retrospective chart review  2006-2014 | 97 children† (175 procedures); 59 boys, 38 girls  Aged 2 months-18 years (mean age 8.6 years; median age 8.8 years)  Primary diagnosis (some children had multiple underlying conditions): CP (n=39), epilepsy (n=25), encephalopathy (n=25), developmental delay (n=13), chromosome abnormalities (n=5) | Free-hand, topical anaesthesia in 75%, general anaesthesia in 25%  Botox®  35U/submandibular gland, 15U/parotid gland | Not reported, case notes were used | Subjective (parental impression of difference in symptoms) | Age  Diagnosis  Epilepsy  Feeding | 22 (13% of all procedures)  Dysphagia (n=18) with 6 children requiring nasogastric feeding and hospitalisation, thickened secretions which did not require management (n=3), difficulty turning head which resolved spontaneously (n=1) |
| Nordgarden^17^ (Norway), 2012 | Cross-over study  Not reported | 5 children; 4 girls, 1 boy  Aged 10-18 years (mean age ~13.7 years)  Primary diagnosis: CP (n=5)  All children had severe drooling at baseline, defined as drooling that occurred several times per day with DQ ≥50 | UG, general anaesthesia  Botox®  100U | Baseline,  8 weeks | Objective (DQ*, salivary flow rate*)  VAS* for QoL | Age  Diagnosis  Baseline drooling | 2 (40%)  Dysphagia leading to reduced intake of food and fluids, which resulted in weight loss and took several weeks to resolve (n=1),  increased speech difficulties persisting for approximately 2 months (n=1) |
| Ong^18^ (Malaysia), 2009 | Prospective open label trial  2007-2008 | 21 children; 9 boys, 12 girls  Aged 4-12 years (mean age 8.4 years; SD 2.5 years)  Primary diagnosis: spastic CP (n=16), dyskinetic CP (n=2), ataxic CP (n=3)  All children had significant drooling (DSFS score ≥6) at baseline | UG, sedation  Botox®  15U/parotid gland, 15U/submandibular gland for <15 kg, 20U/submandibular gland for 15-25 kg, 25U/submandibular gland for >25 kg | Baseline,  2 weeks,  8 weeks,  16 weeks | Objective (DQ*)  Subjective (DSFS*, number of bib changes*, VAS* for drooling severity, caregiver satisfaction scale)  QoL and caregiver issues questionnaire* | Age  Diagnosis  + CP subtype  Epilepsy  Mobility  Baseline drooling (DSFS) | Total adverse event rate not reported.  Pain and swelling (n=4), excessively thick saliva with halitosis (n=2), fever (n=2), difficulty chewing (n=2). All were encountered in the first 2 weeks post-injection and were transient |
| Reid^19^ (Victoria, Australia), 2008 | Multicentre, open-label RCT‡ (BoNT-A vs. no intervention)  2004-2006 | 24 children; 10 boys, 14 girls.  Aged 6-18 years (mean age 11y 0m; SD 3y 5m)  Primary diagnosis: CP (n=13), intellectual disability (n=9), undiagnosed neurological condition (n=1), developmental coordination disorder (n=1)  All children had significant drooling at baseline, mean DIS score 56.5 | UG, general anaesthesia  Botox®  25U/gland or 1U/kg/gland for <25 kg | Baseline,  1 month,  2-3-4-5-6 months,  1 year | Impact (DIS* and shortened version of DIS (severity/ frequency/number of bibs)) | Age  Diagnosis  Epilepsy  Feeding  Speech  Cognition  Mobility  Baseline drooling | 4 (17%)  Thicker, more viscous saliva (n=4), difficulties with swallowing, choking, and deterioration of speech for the first 5 days post-procedure (n=1), severe chest infection (n=1), seizure 2 days post-procedure (n=1)  Increased difficulty with swallowing food or reluctance to eat hard and dry foods in some children (not further specified) |
| Reid^20^ (Victoria Australia), 2013 | Prospective study  2009-2010 | 26 children; 14 boys, 12 girls  Aged 6-18 years (mean age 11y 3m)  Primary diagnosis: CP (n=17), intellectual disability (n=8), oral dyspraxia (n=1)  15 children had severe drooling (mean frequency/severity score 8-10), 9 children had moderate drooling (mean score 5-7), 2 children had mild drooling (mean score 1-4) | UG, general anaesthesia  Botox®  25U/gland or 1U/kg/gland for <25 kg | Baseline,  4 weeks,  2-3-4-5-6 months | Impact (DIS* and severity/ impact items of DIS separately) | Age  Diagnosis  Epilepsy  Feeding  Speech  Cognition  Mobility  Baseline drooling | Total adverse event rate not reported.  Increased problems with swallowing, such as a higher-than-usual frequency of choking on food (n=2) |
| Sales^21^ (Brazil), 2021 | Prospective longitudinal observational cohort study  Not reported | 23 children; 8 boys, 15 girls  Mean age 2y 8m; SD 2.6 months  Primary diagnosis: spastic CP secondary to Congenital Zika Syndrome (n=21), spastic/dystonic CP secondary to Congenital Zika Syndrome (n=2)  All children had severe drooling (DSFS score 7-9) at baseline | Palpation, topical anaesthetic  Dysport® 25U/gland | Baseline,  ±42 days | Subjective (DSFS*, GICS*) | Age  Diagnosis  + CP subtype  Epilepsy  Mobility  Baseline drooling | 2 (9%)  Thickening of saliva and dysphagia lasting for 7 days (n=1), dry cough lasting for 2 days (n=1) |
| Schroeder^22^ (Munich, Germany), 2012 | Prospective cohort study comparing BoNT-A and BoNT-B‡  Not reported | 19 children† (34 procedures); 11 boys, 8 girls  Aged 2.2-23.3 years (mean age 9.8 years) at first BoNT-A session  Primary diagnosis: CP (n=11), syndrome (n=6), leukodystrophy (n=1) suprabulbar palsy (n=1)  All children had severe drooling (DS score ≥4 and DF score ≥3) at baseline | UG, mask anaesthesia  Botox®  Bodyweight dependent dose (42-200U) | Baseline,  4-8 weeks | Subjective (GAS*, DSFS*, number of towel changes per day*)  Parental questionnaire about therapy-related impact on daily life and care | Age  Diagnosis  Baseline drooling | 0 (0%)  No adverse events in BoNT-A group, although information was only available for 13 participants |
| Sürmelioglu^23^ (Adana, Turkey), 2018 | Prospective cohort study  2016-unknown | 27 children; 10 boys, 17 girls  Aged 6-16 years (mean age 11.5 years)  Primary diagnosis: CP (n=27)  All children had profuse drooling at baseline, defined as soiling their clothes and hands (mean VAS severity score 7.9) | No UG, sedation  Botox®  10U/parotid gland  20U/submandibular gland | Baseline,  3 months | Subjective (VAS* for severity and frequency) | Age  Diagnosis  Baseline drooling | 0 (0%)  No complications in any of the patients |
| Taib^24^ (Liverpool, UK), 2019 | Prospective study  2012-2014 | 33 children† (79 procedures); 16 boys, 17 girls  Aged 1-23 years (mean age 11 years)  Primary diagnosis: CP (n=13), syndrome (n=9), other condition (n=9), no relevant diagnosis (n=2)  30 children had severe drooling (DS score ≥4, DF score ≥3) at baseline, 3 children had moderate drooling (DS score 3, DF score 3) | UG, general anaesthesia  BoNT-A formulation not reported  60U divided over the four glands | Baseline,  1 month | Subjective (DSFS*, VAS* for severity, carer-assessed reduction in drooling) | Age  Diagnosis  Epilepsy  GORD  Feeding  Baseline drooling | 1 (1% of all injections)  Dysphagia, resulting in deterioration in eating solid food for six weeks (n=1) |
| Tiigimae-Saar^25^ (Estonia), 2012 | Descriptive prospective study  2010-2011 | 9 children; 4 boys, five girls  Aged 1.6-11 years  Primary diagnosis: spastic CP (n=4), dyskinetic CP (n=5)  Children had moderate to severe drooling (DSFS score ≥3) at baseline (mean DS score 4.3, mean DF score 3.3) | UG, general anaesthesia (n = 7) or topical anaesthesia (n = 2)  Botox® 1.4U/kg/parotid gland  0.6U/kg/ submandibular gland | Baseline,  2-4-6-8-10-12-14-16 weeks | Subjective (DSFS*, VAS* number of bib changes, VAS* number of aspirations)  Change in caregiver’s quality of life | Age  Diagnosis  + CP subtype  Epilepsy  Feeding  Speech Cognition  Head control  Mobility  Baseline drooling (DSFS) | 7 (78%)  Difficulties with swallowing lasting three weeks (n=1), thickening of saliva lasting 8 weeks (n=6) |
| Türe^26^ (Konya, Turkey), 2021 | Prospective cohort study  2017-2018 | 22 children; 12 boys, 10 girls  Aged 2.5-17 years (mean age 7.7 years; SD 4.8 years; median age 5.4 years)  Primary diagnosis: CP (n=14), other neurological disease (n=8)  All children had severe drooling (DS score ≥4) at baseline | UG, anaesthesia not reported  Botox® 1U/kg/gland | Baseline,  1 month | Objective (collected saliva in 24h)  Subjective (DSFS*) | Age  Diagnosis  Baseline drooling (DSFS) | 0 (0%)  No complications in any of the patients |
| Wilken^27^ (Kassel, Germany), 2008 | Randomized trial‡ (BoNT-A vs. BoNT-B)  2003-2006 | 15 children; 7 boys, 8 girls  Aged 1-13 years (mean age 9.8 years)  Primary diagnosis: CP (n=4), Rett syndrome (n=3), Angelman syndrome (n=2), other neurological diagnosis (n=6)  All children had severe drooling (TDS score ≥3) at baseline | UG, no anaesthesia, sometimes topical anaesthetic  Botox®  80U divided over the four glands | Baseline,  4 weeks | Subjective (TDS*) | Age  Diagnosis  Baseline drooling | 5 (33%)  Intermittent problems with swallowing due to viscous saliva resulting in feeding problems (n=4), unilateral parotitis (n=1) |
| Wu^28^ (Taiwan), 2011 | RCT‡ (BoNT-A vs. placebo)  Not reported | 10 children; 3 boys, 7 girls  Mean age 8.6 years; SD 4.1 years  Primary diagnosis: CP (n=10) | UG, anaesthesia not reported  Botox®  30U for <15 kg, 40 U for 15-25 kg, 50 U for >25 kg | Baseline,  1 month,  3 months | Objective (saliva weight*)  Subjective (5-point scale for drooling severity and bib changes) | Age  Diagnosis  Epilepsy Mobility | 0 (0%)  All participants tolerated the procedure well and reported no adverse events |

*Existing scale or questionnaire (as opposed to outcome measure created for this specific study); †Multiple procedures per patient. ‡Only characteristics of the BoNT-A treatment group are displayed. Abbreviations: BoNT-A, botulinum neurotoxin type-A; BoNT-B, botulinum neurotoxin type-B; CP, cerebral palsy; DF, drooling frequency component of the Drooling Severity and Frequency Scale; DIS, Drooling Impact Scale; DS, drooling severity component of the Drooling Severity and Frequency Scale; DSFS, Drooling Severity and Frequency Scale; DQ, Drooling Quotient; DQ5, 5-minute Drooling Quotient; GORD, gastro-oesophageal reflux disease; m, months; mTDS, modified Teacher Drool Scale; RCT, randomized controlled trial; TDS, Teacher Drool Scale; U, units; UG, ultrasound guidance; VAS, visual analogue scale; QoL, quality of life; y, years.

**Table S3.** Response rates and definitions

| **Studies concerning submandibular injections** | | |
| --- | --- | --- |
| **Reference** | **Response rate** | **Definition** |
| Bekkers | 53.8% | *Clinically significant response*: ≥50% reduction in DQ or VAS at 8 weeks post-injection |
| Ciftci | 40% | *Significant response to treatment*: ≥2 points reduction in TDS at 4 weeks post-injection |
| Gok | Not reported |  |
| Hay | Not reported |  |
| Mahadevan | 60.4% | *Improvement correlating to caregiver impression of improvement*: ≥2 points reduction in summed DSFS for more than 4 weeks |
| Van Hulst | 70% | *Clinically relevant response*: ≥50% reduction in DQ and/or ≥2 SDs reduction in VAS at 8 weeks post-injection |
| **Studies concerning four-gland injections** | | |
| **Reference** | **Response rate** | **Definition** |
| Alvarenga | 76.5% or 70.6% | No definition provided, patients with any reduction in DSS or DFS reported |
| Banerjee | 90% | No definition provided, patients with definite reduction and/or complete cessation of drooling at 4 weeks reported |
| Bernardo | Not reported |  |
| Berweck | 70.9% or 63.0% in the 6-17 year old group | No definition provided, patients with at least +1 point (“minimally improved”) on the global impression of change scale at 4 weeks or 16 weeks post-injection reported |
| Gubbay | 60.0%-86.7% | No definition provided, children with any improvement in drooling frequency, effect on the child, effect on the family, bibs and shirts, skin irritation, or coughing/choking after treatment reported |
| Ghazavi | Not reported |  |
| Lungren | 68% | *Clinical efficacy*: at least partially effective (persistence of minimal drooling, partial improvement of quality of life, insufficient improvement in relation to the patient’s or clinician’s expectations) |
| Meece | 100% | No definition provided, children with any reduction in drooling reported |

| Montgomery | 62% or 70% | *Effectiveness of response*: effective (parents reported that their child’s symptoms were obviously and significantly improved) or at least partially effective (the child or their parents had noticed an appreciable difference, but not full resolution of symptoms) |
| --- | --- | --- |
| Nordgarden | 60% or 100% or 80% | No definition provided, children with any reduction in DQ, reduction in saliva secretion, or reduction in the influence of drooling on quality of life reported |
| Ong | 23.8% | No definition provided, children with DQ of 0 at week 16 post-injection reported |
| Reid (2008) | 66.7% | *Meaningful response*: ≥20 point reduction in DIS at 1 month post-injection |
| Reid (2013) | 73.1% | *Clinically important difference*: ≥30 points reduction in DIS at 1 month post-injection |
| Sales | 78.3% | *Therapeutic response*: ≥1 point reduction in DSFS |
| Schroeder | 34% | *Desired reduction with meaningful impact on daily activities*: score ≥0 on goal attainment scale |
| Surmelioglu | Not reported |  |
| Taib | 90.9% | No definition provided, children with any reduction in drooling at 1 month post-injection reported (at least partially effective intervention) |
| Tiigimae-Saar | Not reported |  |
| Ture | 90.9% | *Significant benefit from the injection*: any decrease in drooling severity and frequency at 1 month post-injection |
| Wilken | 96.7% | No definition provided, children with reduction of TDS score to 1 or 2 reported |
| Wu | Not reported |  |

Abbreviations: DFS, Drooling Frequency Scale; DIS, Drooling Impact Scale; DSFS, Drooling Severity and Frequency Scale; DSS, Drooling Severity Scale; DQ, Drooling Quotient; TDS, Teacher Drool Scale; VAS, visual analogue scale.

**References**

1. Bekkers S, Delsing CP, Kok SE, van Hulst K, Erasmus CE, Scheffer ART, van den Hoogen FJA. Randomized controlled trial comparing botulinum vs surgery for drooling in neurodisabilities. Neurology. 2019;92(11):e1195-e204.

2. Bekkers S, Pruijn IMJ, van der Burg JJW, van Hulst K, Kok SE, Delsing CP, Scheffer ART, van den Hoogen FJA. Surgery versus botulinum neurotoxin A to reduce drooling and improve daily life for children with neurodevelopmental disabilities: a randomized controlled trial. Developmental Medicine & Child Neurology. 2021;63(11):1351-9.

3. Ciftci T, Akinci D, Yurttutan N, Akhan O. US-guided botulinum toxin injection for excessive drooling in children. Diagnostic and Interventional Radiology. 2013;19(1):56-60.

4. Gok G, Cox N, Bajwa J, Christodoulou D, Moody A, Howlett DC. Ultrasound-guided injection of botulinum toxin A into the submandibular gland in children and young adults with sialorrhoea. British Journal of Oral & Maxillofacial Surgery. 2013;51(3):231-3.

5. Hay N, Penn C. Botox(®) to reduce drooling in a paediatric population with neurological impairments: a Phase I study. International Journal of Language & Communication Disorders. 2011;46(5):550-63.

6. Mahadevan M, Gruber M, Bilish D, Edwards K, Davies-Payne D, van der Meer G. Botulinum toxin injections for chronic sialorrhoea in children are effective regardless of the degree of neurological dysfunction: A single tertiary institution experience. International Journal of Pediatric Otorhinolaryngology. 2016;88:142-5.

7. van Hulst K, van der Burg JJW, Jongerius PH, Geurts ACH, Erasmus CE. Changes in severity and impact of drooling after submandibular gland botulinum neurotoxin A injections in children with neurodevelopmental disabilities. Developmental Medicine & Child Neurology. 2020;62(3):354-62.

8. Alvarenga A, Campos M, Dias M, Melao L, Estevao-Costa J. BOTOX-A injection of salivary glands for drooling. Journal of Pediatric Surgery. 2017;52(8):1283-6.

9. Banerjee KJ, Glasson C, O'Flaherty SJ. Parotid and submandibular botulinum toxin A injections for sialorrhoea in children with cerebral palsy. Developmental Medicine & Child Neurology. 2006;48(11):883-7.

10. Bernardo P, Raiano E, Cappuccio G, Dubbioso R, Bravaccio C, Vergara E, Peluso S, Manganelli F, Esposito M. The Treatment of Hypersalivation in Rett Syndrome with Botulinum Toxin: Efficacy and Clinical Implications. Neurology and Therapy. 2019;8(1):155-60.

11. Berweck S, Bonikowski M, Kim H, Althaus M, Flatau-Baque B, Mueller D, Banach MD. Placebo-controlled clinical trial of incobotulinumtoxinA for sialorrhea in children: SIPEXI. Neurology. 2021;02.

12. Ghazavi M, Rezaii S, Ghasemi M, Azin N, Reisi M. Botox injection in treatment of sialorrhea in children with cerebral palsy. American Journal of Neurodegenerative Disease. 2023;12(3):97-102.

13. Gubbay A, Marie Blackmore A. Effects of salivary gland botulinum Toxin-A on drooling and respiratory morbidity in children with neurological dysfunction. International Journal of Pediatric Otorhinolaryngology. 2019;124:124-8.

14. Lungren MP, Halula S, Coyne S, Sidell D, Racadio JM, Patel MN. Ultrasound-guided botulinum toxin type A salivary gland injection in children for refractory sialorrhea: 10-Year experience at a large tertiary children's hospital. Pediatric Neurology. 2016;54:70-5.

15. Meece RW, Fishlock KF, Bayley EW, Keller MS. Ultrasound-guided Botox injections of salivary glands in children with drooling. Journal of Radiology Nursing. 2010;29(1):20-4.

16. Montgomery J, McCusker S, Hendry J, Lumley E, Kubba H. Botulinum toxin A for children with salivary control problems. International Journal of Pediatric Otorhinolaryngology. 2014;78(11):1970-3.

17. Nordgarden H, Osterhus I, Moystad A, Asten P, Johnsen UL, Storhaug K, Loven JO. Drooling: are botulinum toxin injections into the major salivary glands a good treatment option? Journal of Child Neurology. 2012;27(4):458-64.

18. Ong LC, Wong SW, Hamid HA. Treatment of drooling in children with cerebral palsy using ultrasound guided intraglandular injections of botulinum toxin A. Journal of Pediatric Neurology. 2009;7(02):141-5.

19. Reid SM, Johnstone BR, Westbury C, Rawicki B, Reddihough DS. Randomized trial of botulinum toxin injections into the salivary glands to reduce drooling in children with neurological disorders. Developmental Medicine & Child Neurology. 2008;50(2):123-8.

20. Reid SM, Walstab JE, Chong D, Westbury C, Reddihough DS. Secondary effects of botulinum toxin injections into salivary glands for the management of pediatric drooling. Journal of Craniofacial Surgery. 2013;24(1):28-33.

21. Sales HF, Cerqueira C, Vaz D, Medeiros-Rios D, Armani-Franceschi G, Lucena PH, Sternberg C, Nobrega AC, Luz C, et al. The impact of botulinum toxin type A in the treatment of drooling in children with cerebral palsy secondary to Congenital Zika Syndrome: an observational study. Neurological Research. 2021;43(1):54-60.

22. Schroeder AS, Kling T, Huss K, Borggraefe I, Koerte IK, Blaschek A, Jahn K, Heinen F, Berweck S. Botulinum Toxin Type A and B for the reduction of hypersalivation in children with neurological disorders: A focus on effectiveness and therapy adherence. Neuropediatrics. 2012;43(1):27-36.

23. Surmelioglu O, Dagkiran M, Tuncer U, Ozdemir S, Tarkan O, Cetik F, Kiroglu M. The effectiveness of botulinum toxin type A injections in the management of sialorrhea. Turkish Archives of Otorhinolaryngology. 2018;56(2):111-3.

24. Taib BG, Williams SP, Sood S, Ung K, Nixon PP, Sharma R. Treatment of sialorrhoea with repeated ultrasound-guided injections of botulinum toxin A into the parotid and submandibular glands. British Journal of Oral & Maxillofacial Surgery. 2019;57(5):442-8.

25. Tiigimae-Saar J, Leibur E, Kolk A, Talvik I, Tamme T. Use of botulinum neurotoxin A in uncontrolled salivation in children with cerebral palsy: a pilot study. International Journal of Oral & Maxillofacial Surgery. 2012;41(12):1540-5.

26. Türe E, Yazar A, Dundar MA, Bakdik S, Akin F, Pekcan S. Treatment of sialorrhea with botulinum toxin A injection in children. Nigerian Journal of Clinical Practice. 2021;24(6):847-52.

27. Wilken B, Aslami B, Backes H. Successful treatment of drooling in children with neurological disorders with botulinum toxin A or B. Neuropediatrics. 2008;39(4):200-4.

28. Wu KP, Ke JY, Chen CY, Chen CL, Chou MY, Pei YC. Botulinum toxin type A on oral health in treating sialorrhea in children with cerebral palsy: a randomized, double-blind, placebo-controlled study. Journal of Child Neurology. 2011;26(7):838-43.
